# Systemic Factors Associated with Treatment Response in Diabetic Macular Edema

**DOI:** 10.1155/2020/1875860

**Published:** 2020-03-19

**Authors:** Wendy Meihua Wong, Caroline Chee, Mayuri Bhargava, Charmaine Chai, Hazel Lin, Paul Zhao, Erlangga Ariadarma Mangunkusumo, Thet Naing, Yew Sen Yuen, Tien Yin Wong, Xinyi Su, Gopal Lingam

**Affiliations:** ^1^National University Hospital, Singapore; ^2^Singapore Eye Research Institute, Singapore; ^3^National University of Singapore, Singapore; ^4^Institute of Molecular and Cell Biology, A∗STAR, Singapore

## Abstract

**Purpose:**

To identify systemic factors that may influence the response to anti-VEGF therapy in patients with diabetic macular edema (DME).

**Methods:**

35 patients undergoing anti-VEGF injections for centre-involving DME were studied in this prospective observational study. The primary outcome was change in macular thickness one month after treatment, measured using spectral-domain optical coherence tomography (OCT). At baseline, information on various systemic factors was collected including glycosylated hemoglobin (HbA1c), serum VEGF levels, lipid profile and markers of renal function, and blood pressure. Thirty-three of the 35 patients were included in this study. Nonparametric statistical tests were used for the analysis of the data in view of the nonnormal distribution of the outcome variables. Multivariate analysis was performed using logistic regression. Stata 12.1 software was used for the analysis. *Main Outcome Measures*. Reduction in macular central subfield thickness (on spectral-domain OCT) and change in logMAR visual acuity at one month after injection.

**Results:**

Lower HbA1c levels (7% or less) were significantly associated with greater reduction in central macular subfield thickness at one month after injection of bevacizumab or ranibizumab on both univariate analysis (*p*=0.012) and multivariate analysis (*p*=0.012) and multivariate analysis (

**Conclusions:**

Better glycemic control is associated with a greater reduction in central macular thickness after the first injection of bevacizumab or ranibizumab in diabetic macular edema. Patients with high levels of HbA1c and poor response to anti-VEGF may benefit from strict control of their blood glucose.

## 1. Introduction

Diabetic macular edema (DME) is a vision-threatening complication of diabetes. In DME, accumulation of fluid in the macula results in loss of central vision, which is important for facial recognition, reading, and driving. DME affects 1 in 15 people with diabetes [1] and is the leading cause of blindness in young adults in developed countries [2].

Intravitreal injections of antivascular endothelial growth factor (anti-VEGF) have revolutionized the treatment of patients with DME, causing visual impairment. Several landmark studies have demonstrated that anti-VEGF therapy, compared to laser photocoagulation, provides superior visual outcomes [3, 4]. In the Diabetic Retinopathy Clinical Research Network Protocol T, three commonly used anti-VEGF agents, bevacizumab, ranibizumab, and aflibercept, were shown in the randomized controlled trial to improve vision in centre-involving DME [5].

Despite the proven benefits of anti-VEGF therapy, a subgroup of patients has persistent DME after an initial course of anti-VEGF therapy. A secondary analysis of Protocol T showed that after six monthly intravitreal anti-VEGF injections, persistent macular thickening was present in 65.6%, 41.5%, and 31.6% of eyes treated with bevacizumab, ranibizumab, and aflibercept, respectively [6]. The clinical challenge of predicting individual response to anti-VEGF therapy remains. Being able to do so will be invaluable for the physician to counsel patients and manage expectations.

The influence of systemic factors on the occurrence of diabetic retinopathy and other micro- and macrovascular complications has been well studied. Studies have shown that tight control of blood sugar and other associated systemic factors such as hypertension, serum cholesterol, and kidney function can significantly delay the onset of diabetic retinopathy [7–11]. However, it is not known if these systemic factors affect the anatomical and visual response to anti-VEGF intravitreal injections.

In this prospective study, we explored whether systemic factors, such as blood pressure, glucose control, cholesterol, triglyceride, and creatinine levels at the time of intravitreal anti-VEGF injection, affect the visual or anatomic response at one month after initiating the treatment.

## 2. Materials and Methods

### 2.1. Study Design

This prospective, single-centre, observational study was conducted with Institutional Review Board approval and adhered to the tenets of the Declaration of Helsinki. Informed consent was obtained from all study participants. Eligible participants had centre-involved DME confirmed on spectral-domain optical coherence tomography (OCT) (Spectralis HRA + OCT, Heidelberg Engineering, Heidelberg, Germany). Patients who had prior vitreoretinal surgery, laser, or anti-VEGF injections to the study eye within 2 months or were unable to come for review one month after the injection were excluded. The study recruited consecutive patients who required anti-VEGF for treatment of DME and were able to provide informed consent.

### 2.2. Assessment of Systemic and Metabolic Parameters

The following baseline clinical characteristics were recorded: age; gender; duration of diabetes; diabetic medications; and associated systemic conditions such as hypertension, nephropathy, and ischemic heart disease.

On the day of injection, blood was collected to check the glycosylated hemoglobin (HbA1c) and serum VEGF levels, lipid profile (triglyceride, total cholesterol, and fractions), and markers of renal function (estimated glomerular filtration rate (eGFR) and serum creatinine). The brachial systolic and diastolic blood pressures (BP) were recorded twice with a digital manometer, at intervals of 10 minutes, with the lower of the two recordings taken as the final value.

### 2.3. Assessment and Treatment of DME

The Snellen best corrected visual acuity (BCVA) was recorded. The central subfield thickness (CST) was measured on spectral-domain optical coherence tomography (OCT). The change in BCVA and CST, between baseline and one month after IVT anti-VEGF, was used to assess the functional and morphological response to treatment, respectively. Study participants received either intravitreal bevacizumab (1.25 mg in 0.05 ml) or ranibizumab (0.5 mg in 0.05 ml).

### 2.4. Statistical Analysis

The Snellen BCVA was converted to LogMAR units and the ETDRS letter score for statistical analysis.

Continuous variables were dichotomised as normal and abnormal. The value for dichotomisation was based on published literature (>140 mmHg for systolic BP [12]; >90 mm/hg for diastolic blood pressure [5]; >7.0% for HbA1c [13]; and >308 pg/mL for serum VEGF levels [14]) or the laboratory-specific reference range (>5.2 mmol/litre for cholesterol; >2.2 mmol/litre for triglycerides; >3.3 mmol/litre for LDL; <1 mmol/litre for HDL; >3.5 for total cholesterol : HDL ratio; >120 *μ*mol/litre for serum creatinine; and <90 ml/min/1.73 m^2^ for eGFR).

Univariate analysis was performed with nonparametric tests as the distribution of the outcome variables were significantly skewed to the right. Evaluation of the effect of each of the systemic factors (normal vs abnormal) on the change in CST and BCVA was performed with Mann–Whitney *U* test. Spearman correlation test was performed for testing correlation between linear variables such as visual acuity and central subfield thickness. Multivariate analysis was performed using logistic regression analysis and stepwise backward selection of variables to be included in the final model. The Strata 12.1 software was used for statistical analysis.

## 3. Results

### 3.1. Baseline Characteristics

Over a one-year period, 35 eyes of 35 participants received either intravitreal bevacizumab (*n* = 25, 71.4% of eyes) or ranibizumab (*n* = 10, 28.6% of eyes). Data were analyzed for 33 eyes that completed the one-month follow-up visit.

The baseline demographic and study eye characteristics are summarized in Table 1. The mean duration of diabetes for study participants was 11.8 ± 9.5 years. The mean baseline CST was 440.5 ± 136.3 microns. There was no statistically significant difference in the mean baseline CST of patients with HbA1c ≤7.0% and patients with HbA1c >7.0% (*p*=0.27).

The systemic and metabolic factors at time of anti-VEGF treatment are shown in Table 2. The serum HbA1c was greater than 7.0% in 57.1% of participants.

No correlation was found between the baseline CST and BCVA (Spearman correlation test).

### 3.2. Effect of Treatment on Visual Acuity

The final visual acuity was 6/12 (70 letters) or better in 51.4%; >6/60 to <6/12 (36 to 69 letters) in 34.3%; and less than or equal to 6/60 (35 letters) in 8.6%. The visual acuity was unchanged in 12 eyes (36.4%). The visual acuity improved in 11 eyes (33.3%), with an increase in the visual-acuity letter score ranging from 3 to 35 letters. An improvement of ≥15 letters was observed in 2 eyes (18.2%). The visual acuity worsened in 10 (30.3%) eyes, with 3 eyes (30%) having a ≥15 letters decline in the visual-acuity letter score.

### 3.3. Effect of Treatment on Retinal Thickening

At 4 weeks after injection, the CST decreased, on average by 82.03 ± 150.19 microns (range: −519 *μ*m to + 138 *μ*m). By percentage (with reference to baseline) the change ranged from −65.6% to +28.9%. The Spearman correlation test did not reveal any correlation between the change in the level of vision and the change in CST.

### 3.4. Association of Systemic Factors with Anatomical and Visual Response

Tables 3 and 4 summarize the results of univariate and multivariate analysis of influence of various independent variables on the outcome variables.

On univariate analysis, only the HbA1c level was significantly associated with reduction of CST after anti-VEGF treatment (*p*=0.012). The mean reduction in CST was 130 *μ*m in the group with HbA1c ≤7.0% and 41.9 *μ*m in the group with HbA1c >7.0%. On multivariate logistic regression analysis, the HbA1c level was associated with reduction in CST after anti-VEGF therapy (odds ratio −0.019, 95% confidence interval 0.042 to 0.944). The serum levels of VEGF had a moderate correlation with the reduction of CST, but this difference did not achieve statistical significance (*p*=0.1894).

The change in BCVA after treatment did not have any correlation with the systemic factors that were tested.

## 4. Discussion

In the management of diabetic macular edema, following several landmark trials [3, 12, 13], anti-VEGF therapy has become the standard of care. However, a subgroup of patients lacks “good” visual or anatomical response for unclear reasons. Postulated factors include local factors, such as poor retinal pigment epithelium health. In this study, we hypothesized that systemic factors have an important role in the clinical response to anti-VEGF treatment.

### 4.1. Association of Systemic Factors with Anatomical Response after Treatment

Our study has identified that HbA1c levels of 7% or less, at the time of intravitreal anti-VEGF injection, is associated with a better anatomical response, as assessed by the reduction in CST on OCT. This suggests that tight glucose control during the treatment period is important for good clinical outcome and is consistent with previous studies [14, 15].

We also hypothesized that serum VEGF levels might reflect intraocular VEGF levels and thus predict the anatomical response to intravitreal anti-VEGF injections. Although a statistically significant difference was not found (*p*=0.1894), our results suggest a trend towards better anatomical response with lower serum VEGF levels.

An earlier study found serum creatinine and cholesterol levels to correlate with reduction in CST after treatment [16]. In this study, the serum creatinine and glomerular filtration rate (eGFR) did not show an association with CST after anti-VEGF therapy. Additionally, patients on dialysis did not show a preferential lack of response to treatment, although our study may not be sufficiently powered to address this.

### 4.2. Association of Systemic Factors with Visual Outcome after Treatment

Our study showed a significant association between lower HbA1c and CST reduction, but a similar association was not found for BCVA. However, changes in the CST and the visual acuity do not necessarily correlate. In the DRCR.net Protocol I, the CST and VA of eyes treated with laser had a modest correlation [17]. In the DRCR.net Protocol T, the change in CST at 12 weeks and visual acuity at 2 years did not have a strong association [18].

There is conflicting evidence on correlation of HbA1c and visual response to anti-VEGF from large phase 3 trials [19, 20]. An analysis of ranibizumab-treated patients from the RISE and RIDE trials did not find an association between mean change in BCVA at weeks 52 and 100, with the baseline HbA1c [19]. This is in contrast to an analysis of aflibercept-treated patients from the VISTA and VIVID trials, which found that the mean improvement in VA at 2 years was dependent on HbA1c levels [21]. More recently, an exploratory analysis of DRCR.net Protocol T, in which participants were randomized to receive bevacizumab, ranibizumab, or aflibercept, similarly found the magnitude of vision improvement after anti-VEGF treatment to be associated with HbA1c levels [20].

One possible explanation for the discrepancy between studies is that patients with similar HbA1c levels can have marked differences in their daily glucose profiles, with variable frequency and duration of glucose excursions [22, 23]. Transient hyperglycemic spikes can be a HbA1c-independent risk factor for diabetes-related complications, due to transient episodes of oxidative stress [24]. Most studies have used HbA1c levels measured at the time of injection which reflects the blood glucose control in the previous 2 months and not prospectively after administering treatment. This could also be a limitation in understanding the correlation between HbA1c levels and response to anti-VEGF treatment.

### 4.3. Study Strengths and Limitations

The principal strength of this study is the prospective evaluation of the impact of other comorbidities on the short-term anatomical or visual response to anti-VEGF treatment. There are several limitations to this study, including the small sample size and inclusion of study participants receiving different anti-VEGF agents.

## 5. Conclusion

Although HbA1c has been demonstrated to be a marker and strong predictor of vascular complications in diabetic patients [7], its prognostic significance during treatment of DME and its effect on the efficacy is not clear. In our study, we identified that good glycemic control, as defined by an HbA1c level of less than 7%, in the period preceding anti-VEGF treatment, is associated with greater reduction in central subfield thickness on macular OCT. This has significant implications for our clinical management of DME patients with suboptimal response to initial anti-VEGF therapy. If the HbA1c levels are high in these patients, one can enforce rigid control of blood glucose, continue with the same therapy, and reassess, rather than switch to a different drug. This is because the initial lack of optimal response might be due to the lack of proper blood glucose control. Our results also will help with patient counselling and management of their expectations after their first intravitreal anti-VEGF injection.

## Figures and Tables

**Table 1:** Patient demographics, clinical, and ocular characteristics (*n* = 35).

Parameter			Number (percentage)
Demographics	Gender	Male	17 (48.6)
		Female	18 (51.4)
	Age	Mean/SD	62.1 yrs/SD-7.4
		Range	50–80 yrs

Treatment for diabetes mellitus	Oral hypoglycemic agents		20 (57.14)
	Only insulin		4 (11.4)
	Insulin + oral hypoglycemic agents		11 (31.4)

Comorbidities	Hypertension		34 (97.1)
	Ischemic heart disease		11 (31.4)
	Nephropathy		17 (48.6)
	On renal dialysis		3 (8.6)

Ocular features	Snellen best corrected visual acuity	6/12 or better	21 (60)
		>6/60 to <6/12	11 (34)
		≤6/60	3 (8.6)
	Lens status	Minimal cataract	19 (54.3)
		Significant cataract	8 (22.9)
		Pseudophakia	8 (22.9)
	Proliferative diabetic retinopathy	2 (5.7)	

Prior treatment for diabetic retinopathy/maculopathy	Previous laser	PRP	18 (51.4)
		Macular	6 (17.1)
		Both	2 (5.7)
	Prior intravitreal anti-VEGF therapy		23 (65.7)

SD, standard deviation; PRP, pan retinal photocoagulation; anti-VEGF, antivascular endothelial growth factor.

**Table 2:** Prevalence of abnormal parameters related to systemic condition (*n* = 35).

S/N	Parameter	Number (percentage)
1	Systolic blood pressure >140 mm/hg	23 (65.7)
2	Diastolic blood pressure >90 mm/hg	4 (11.4)
3	Serum creatinine >120 *μ*mol/litre	16 (45.7)
4	eGFR <90 ml/min/1.73 m^2^	25 (71.4)
5	Serum total cholesterol >5.2 mmol/L	10 (28.6)
6	Serum triglycerides >2.2 mmol/L	13 (37.1)
7	Serum high density lipoproteins <1 mmol/L	6 (17.1)
8	Serum low-density lipoproteins >3.3 mmol/L	9 (25.7)
9	Ratio of LDL to total cholesterol >3.5	21 (60)
10	Serum HbA1c >7%	20 (57.1)
11	Serum VEGF levels >308 pg/mL	24 (68.6)

eGFR, estimated glomerular filtration rate; LDL, low-density lipoproteins; HbA1c, glycosylated hemoglobin; VEGF, vascular endothelial growth factor.

**Table 3:** Association of various systemic factors with change in central subfield thickness (CST) and change in logMAR visual acuity (*N* = 33), (Mann–Whitney *U* test).

S/N	Systemic factor		Reduction in CST		*p* value	Change in logMAR visual acuity		*p* value
			Mean	SD		Mean	SD	
1	IHD	No (*n* = 23)	98.43	165.38	0.3371	0.013	0.239	0.7479
		Yes (*n* = 10)	44.3	105.23		0.006	0.193	

2	On dialysis	No (*n* = 30)	77.63	145.77	0.7542	0.006	0.234	0.2105
		Yes (*n* = 3)	126	222.7		0.06	0.053	

3	Systolic BP	≤140 (*n* = 11)	71.73	180.23	0.4337	−3.05	0.29	0.09532
		>140 (*n* = 22)	87.18	137.18		0.016	0.192	

4	Diastolic BP	≤90 (*n* = 29)	79.31	155.30	0.6994	0.283	0.228	0.1492
		<90 (*n* = 4)	101.75	122.09		−0.115	0.160	

5	Creatinine	≤120 (18)	77.61	134.9	0.6255	−0.008	0.212	0.2582
		<120 (*n* = 15)	87.3	171.47		0.033	0.243	

6	eGFR	>90 (*n* = 8)	82.13	172.0	0.8831	0.058	0.267	0.7961
		<90 (*n* = 25)	82	146.44		−0.004	0.212	

6	Total cholesterol	≤5.2 (*n* = 24)	72.67	147.39	0.7464	−0.018	0.197	0.2905
		>5.2 (*n* = 9)	107.25	163.7		0.087	0.283	

7	Triglycerides	</ = 2.2 (*n* = 22)	91.14	166.77	0.9239	0.054	0.244	0.1645
		>2.2 (*n* = 11)	63.82	114.99		−0.075	0.153	

8	HDL cholesterol	≥1 (*n* = 27)	69.67	134.7	0.7794	0.002	0.208	0.6322
		<1 (*n* = 6)	137.67	213.2		0.05	0.307	

9	LDL cholesterol	</ = 3.3 (*n* = 25)	69.76	145.02	0.5015	−0.017	0.192	0.2627
		>3.3 (*n* = 8)	120.38	169.67		0.098	0.301	

10	LDL: total cholesterol	≤3.5 (*n* = 14)	41.86	75.49	0.8841	−0.054	0.157	0.5558
		>3.5 (*n* = 19)	111.63	183.86		0.059	0.256	

11	HbA1c	≤7 (*n* = 15)	130.13	158.44	**0.012**	0.001	0.259	0.8821′
		>7 (*n* = 18)	41.94	134.32		0.019	0.197	

12	Serum VEGF	≤308 (*n* = 10)	41.1	132.49	0.1894	0.008	0.065	0.6879
		>308 (*n* = 23)	99.83	156.64		0.012	0.267	

CST, central subfield thickness; IHD, ischemic heart disease; BP, blood pressure; eGFR, estimated glomerular filtration rate; HDL, high-density lipoprotein; LDL, low-density lipoproteins; HbA1c, glycosylated hemoglobin; VEGF, vascular endothelial growth factor.

**Table 4:** Multivariate logistic regression analysis using stepwise backward selection for influence of various factors on reduction in central macular thickness with anti-VEGF injection.

S/N	Parameter	Odds ratio	*p* value	Confidence interval	
				Lower	Upper
1	HbA1c	0.019	**0.042**	0.042	0.944
2	LDL: total cholesterol	3.19	0.172	0.603	16.83

Other factors were dropped during the stepwise backward selection.

## Data Availability

The data used to support the findings of this study are available from the corresponding author upon request.
